# Hospital Meetings

**Published:** 1906-03-24

**Authors:** 


### HOSPITAL MEETINGS.

### CHARING CROSS HOSPITAL.

### SPECIAL APPEAL FOR AN EMERGENCY FUND.

The Earl of Kilmorey presided at a public meeting held in His Majesty's Theatre on Thursday, March 15, on behalf of an emergency fund to pay off the debt on the Charing Cross Hospital, for which £100,000 is required. The Duchess of Albany occupied the Royal box.

In opening the proceedings Lord Kilmorey said that as the recently elected chairman of Charing Cross Hospital it was his duty to preside over that gathering, but he wished that duty had fallen into more experienced hands and to a more eloquent speaker; but he felt sure that the eloquence and persuasive power of those who would follow him would amply make amends for his shortcomings. He would begin by giving them a short history of the hospital. It stood in the busiest quarter of London and was receiving an average of from 20,000 to 21,000 out-patients and 2,500 in-patients every year, and he thought they would agree that if it were suddenly to disappear and leave a vacancy it would be hard, if not impossible, to fill, it would be nothing short of a metropolitan disaster; their meeting there that day was to take steps to avert such a catastrophe. Although now possessing 187 beds in almost daily occupation, very few being ever empty, and then only for a few hours at a time, it was originally a small dispensary, founded by the late Dr. Golding in 1818. It was started for the purpose of affording good medical and surgical advice and drugs, free of cost, to the sick poor in the neighbourhood of Charing Cross, and was so maintained by voluntary contributions for fifteen years. In 1833 it became evident that the dispensary was inadequate to supply all the relief needed, and a plot of ground was therefore purchased, being part of the present site, and thereon a substantial building was erected, which was subsequently known as Charing Cross Hospital. The scheme of the originators of the hospital embraced the foundation and maintenance of a medical school; and except by means of a special Act of Parliament the responsibilities undertaken by their affiliation with that useful and beneficent institution could not be avoided.

Fifty years later, in 1883, when they had as president the late Duke of Saxe-Coburg-Gotha, better known in this country by his original title of Duke of Edinburgh, R.N., the committee came to the conclusion that it would be advisable to petition for a Royal Charter, and on September 24 their charter of incorporation was granted by her late Majesty Queen Victoria. Then twenty years more rolled by, at the end of which it was seen that the hospital was gradually becoming inadequate for the needs of the district. Plans for rebuilding and enlarging the premises were completed; large sums of money were collected, more land was acquired, and the huge building as it now stood was erected and equipped, being now three times as large as that originally built on the site in 1818. But this was the commencement of their sorrows and the origin of their tears. The heavy expense incurred in bringing the hospital into an up-to-date condition far exceeded the estimates, and not only was much left undone for want of funds, but the carrying out of what was completed left them deeply in debt. They were constrained therefore to entreat the public's kind assistance. They had now a hospital which was second to none, and this he said with natural pride as its new chairman, and without fear of contradiction. Its extraordinary popularity was shown by the steady inrush of patients from all parts of London and from the provinces. It would be a lamentable calamity if they were obliged to put up their shutters. That popularity was the result of the widespread reputation of the medical, surgical, and nursing treatment enjoyed within its walls. Time was when their premises and appliances were out of date, but the advance of professional science and the corresponding development of nursing, as well as modern sanitary reforms and the improvements in antiseptic treatment had compelled them to undertake the expense of rebuilding and extension and equipment; and while they had got most valuable results they had also been plunged into a morass of difficulty and debt. Improvements and reforms were unavoidable but costly necessities. But they had never yet made an appeal in vain, and he refused to believe it would be so now. What was wanted was steady and immutable subscriptions, not large sums at a time, from everyone in the district so that they would be able to reckon on how much their income would be, and so decide how much they could do.

### Its Unique Site in the Largest Empire City, where all Classes Receive Benefits.

The Bishop of London then moved : "That the Charing Cross Hospital, occupying a unique position in the centre of the largest city of the world, is deserving of the most generous support of all classes, and that this meeting pledges itself to use every endeavour to raise an Emergency Fund of £120,000 to pay off the debt and meet the annual deficit which endanger the continued maintenance of the hospital."

He should like to call attention, he said, to three expressions contained in the resolution. The first was the reference to the unique position. His East-end training in debate always put him in the frame of mind of wanting to knock someone down—in argument of course—(laughter)—and on this occasion he pictured to himself someone who would put forward the proposition that Charing Cross Hospital was not wanted. He would say, probably, that the sick poor had largely withdrawn from the neighbourhood. This was not wholly true, but his retort would be, Is there anywhere else to be found a single site like Charing Cross? Why, it was the centre of the traffic of the world! He would say nothing of the cabs which brought people to be entertained and interested by their friend Mr. Beerbohm Tree. (Applause.) He would say nothing of the wains and wagons which toiled along the converging streets; but when to these was added the danger of the motor buses—(laughter)—he was bound to ask, Is it safe to have Charing Cross without Charing Cross Hospital? As well might they have a boiler without a safety valve. (Hear, hear.) He hoped he had knocked his first man down! (Laughter). The second phrase he would deal with was "the largest city in the world." No one had more cause to realise the size of London than the Bishop of London. On that side of the water they had an increase in the population of 40,000 a year, and his brother of Southwark he knew had about the same; and when London was increasing by 80,000 people a year was that a time to ask them to shut up a hospital? (Hear, hear.) The Bishops knew how much had to be done in providing churches and clergy for the rapidly-increasing population; and it could hardly be expected that the Church should carry on its back the hospitals as well. In the earlier days of Christianity for a thousand years the house governor of every hospital in Europe was the Bishop of the diocese, and he was therefore proud to raise his voice in that cause, but it was work which all classes should join to support. He hoped the whole community was so trained in Christian principles that it would rise to the opportunity. The third point in the resolution was the reference to "all classes." He would remind them of what had been done in the East-end for the great hospital there, and he could assure them that among the working classes no appeal was more popular or met with so ready a response as that for the hospitals.

That being so should not they, as representing the well-to-do classes, be equally forward in good works. If they did not the only alternative would be for the hospitals to be put on the rates, and he should consider that the Christian spirit of the country had fallen to a low ebb if that took place. They were told that the health-giving breezes were caused by the rush of cold air to the heat centres, and as he looked at the suffering and pain of London and saw it met by kindness and help, he thought of that as a sweet breeze of sympathy which ennobled London and raised the life of the whole community to a higher level. (Applause.)

In the unavoidable absence of Viscount Ridley, Mr. H. A. Harben seconded the resolution and congratulated Charing Cross Hospital on its new chairman. (Cheers.) They knew how much depended on a good chairman, and he looked forward with hope and confidence to a new era of prosperity. He was connected with another hospital and he knew how deep were their needs. The claims of a great general hospital were not only local; their patients came from far and near. They were training grounds for the physicians, the surgeons and the nurses on whom they all depended for health, for comfort, and often for life. It would be shocking for the hospitals to fall on the rates, for such cold charity would be a poor substitute for the warm sympathy and benevolence of the voluntary system. Not only that, but their expenses would be largely increased, for no claim could then be disregarded. It was very hard to have to make constant appeals for money, but if they chastised them with whips, any local authority to whom the control would be given would chastise them with scorpions. Let them, therefore, be wise in time, lest a worse thing come unto them.

The Rev. S. Singer (Jewish Chaplain to the hospital) supported the resolution. He was specially grateful, he said, for that opportunity, because it was a proof, if one were needed, of the absolutely unsectarian character of the work carried on by Charing Cross Hospital as well as by all the great medical charities of the country. He represented one of the smallest sections of the community—not more than half per cent. of the population of London, and not more than 1-10th per cent. of the 400,000,000 souls within the bounds of the Empire. Speaking with an experience of its working of twenty years, he could testify that the spirit of the hospital was one of absolute humanity, and that spirit animated every portion of it, from the head of the administration to the porter at the gate. But perhaps they did not sufficiently bear in mind that the best work done—the work of the highly-skilled physicians and surgeons—was absolutely gratuitous. He considered the suggestion to convert the hospital into one for paying patients as infelicitous and inopportune, especially when it was remembered that King's College Hospital was about to be transferred to South London. Charing Cross Hospital had the reputation of being a most persistent and pertinacious beggar, and he would like to tell them a story to illustrate this. A boy in the suburbs, with the irresponsibility of boyhood, had swallowed several silver coins. A local practitioner was called in and extracted nearly all of them, but one shilling lodged in an inconvenient part of the œsophagus. It was suggested that he should go to a hospital and someone said at once : " Oh ! take him to Charing Cross." "But why Charing Cross ?" it was asked. "Because there is no hospital in London so successful in getting the last shilling out of anybody." (Laughter.) Whether they believed that story or not he hoped they would join with the committee in extracting from the public the last shilling necessary to make up the £120,000 required to place and keep Charing Cross Hospital in a satisfactory position. (Cheers.)

The resolution was then put to the meeting and carried unanimously.

The Chairman mentioned that the Festival Dinner of the hospital would take place at the Hotel Cecil on May 23, Mr. Whitelaw Reid, the American Ambassador, having consented to preside. The donations to the fund already amounted to £6,500. He then moved a vote of thanks to Mr. Beerbohm Tree for the use of the theatre for that meeting. It was only one more instance of the invariable good feeling shown to the hospitals of London by the theatrical profession.

Mr. Tree, in acknowledging the compliment, said he felt it a privilege to be able to welcome them within those walls. It would indeed be a calamity to theatre-land if Charing Cross Hospital became a thing of the past. He could testify to the work done there and to the unvarying courtesy extended to members of his profession. It might be supposed when State Socialism was doing so much to create greater happiness and greater welfare for the people that the endowment of hospitals by the State should be suggested. He hoped it might precede the endowment of the theatre. (Laughter.) When State Socialism was doing so much—when they heard the cry of Free Food for Little Loafers—(laughter)—he meant the children—something of the kind might be expected; but be that as it may, it occurred to him, as they were always on the look out for new devices to raise money, that the ladies might make a house-to-house visitation of the neighbourhood. Every woman—every good woman—was at heart a Socialist, at any rate in social matters, and he thought it would be a capital thing to enlist their services. The hospital was in a very honourable state of bankruptcy, and unlike other concerns a hospital was often most flourishing when deepest in debt. They must however, do their best to get it out, and he was glad to have been able to do something to help so great and historic an institution. (Applause.)

A vote of thanks to the chairman was moved by Mr. Bloxam and seconded by Mr. Malcolm, the treasurer, and the proceedings terminated.

---

### HAMPSTEAD GENERAL HOSPITAL.

Mr. J. S. Fletcher, M.P., presided on Saturday last (17th instant) at the 24th annual general meeting of the Hampstead General Hospital. In moving the adoption of the report and financial statement, he said he did so with special pleasure on that occasion, since, owing to the great generosity of an anonymous benefactor, the Council was in a position to tell them that the hospital now had the funds to complete the building and equipment, and that their business from that time was to secure sufficient subscriptions and donations to pay for its maintenance. This removal of the burden of the Building Fund was a very great relief to the officers. He congratulated them very heartily on the possession of so kind a friend, and that after so many years of effort and growth they were now within measurable distance of the successful completion of a hospital worthy of the district. Glancing through the report, they would notice the reference to the out-patient department. The question of out-patients at hospitals was a very difficult one, and he knew there were many medical men who wished they could do without them altogether. From time to time, unless the most careful precautions were observed, they were taken advantage of by mean people whose circumstances were not such as to justify them in so doing. Personally, he should have been glad if they could have seen their way to do without out-patients, but they had done the next best thing in that they had allied themselves with the dispensaries of the neighbourhood. In a place like Hampstead, with such excellent dispensaries, this was most desirable, and he would like to see preference given to people who were regular subscribers to a dispensary. Every working man nowadays ought to be a subscriber. Their children's education was being paid for; there was a prospect that their food would soon be paid for; and many people seemed to think that their books and clothes ought also to be paid for, and there was no excuse for their not subscribing to a dispensary. He congratulated them that the Zunz trustees had seen their way to give another £3,000 to the building fund, making £4,000 from that source. It was another proof that the institution was well managed, for he understood that those trustees took a serious view of their responsibilities, and made most careful investigations. So also it was gratifying that King Edward's Fund had given them another £2,000, because there was no doubt that that fund was dispensed in the most careful way. The retirement of Dr. Heath Strange from the acting medical staff was another important event of the year. He was the founder of the hospital, and they must all rejoice that he had lived to see it established and hope that he would still live to see it completed and in a thoroughly prosperous condition. He had been invited to become a member of the consulting staff, so that they would not lose the benefit of his advice and assistance. Referring to their paying patients, he thought it was desirable that all hospitals should provide for them. There were, of course, many nursing homes where rich people could go at need, but he was disposed to think that he would rather undergo an operation in a hospital than in a nursing home from the greater sense of security it must give.

Mr. Ernest Collins, the Chairman of the Council, seconded the resolution, and recalled that three years ago not a brick of the new building had been laid, whereas now, thanks to the £20,000 from their anonymous friend, they were not only in occupation, but the cost of building, the mortgage on the land, and the debt to the bank had been paid, and they were on their own freehold and free from debt. They had 58 patients in that day; all the paying beds were full, and all the free beds, with the exception of two or three in the isolation and accident wards. The annual dinner would take place on May 17, when they hoped to get enough to wipe off the deficit of the last two years and get a substantial sum in hand.

The resolution was agreed to; thanks were given to the medical staff and the Ladies' Association, and the Council was re-elected.

In seconding the re-election of Mr. Malcolm as Treasurer, Dr. Heath Strange recalled that of the originators of the scheme only four were now left—Mr. Malcolm, Mr. Mellish, Mr. Owthwaite, and himself.

Dr. Strange was elected as an additional consulting medical officer, with the designation of consulting surgeon, and after the election of the medical staff a vote of thanks to the Chairman closed the proceedings.

### POPLAR HOSPITAL.

The Hon. Sydney Holland presided at the annual meeting of the Poplar Hospital for Accidents on the 14th inst. In moving the adoption of the report and accounts for 1905 he said that that, their jubilee year, had been a very important one in many respects. It had been a record year both in receipts and expenditure, and there were several very satisfactory items on both sides of the accounts. Their total ordinary income was £11,421, as compared with £10,800 last year. They had had to expend some £800 in doing up the out-patient department, but no more would have to be spent on it for a long time, and they would save a considerable amount on the annual cleaning. It was an experiment, the walls being done in a sort of mosaic instead of paint—he believed there was no other like it—but they had every reason to be satisfied with the result. At the dinner that year their President, Mr. Edgar Speyer, took the chair, and they had the largest amount they had ever collected—over £7,000; their hearty thanks were due to him for his efforts. The dock employés, too, had worked very hard, and had raised the record amount of over £860. He must point out that the accounts looked worse than they ought, because the working men's societies' subscriptions did not come in till the beginning of 1906, and so, though collected in 1905, they did not appear in the 1905 accounts. There had been an increase of £1,000 in legacies and of £111 in the amount received from the Hospital Sunday Fund. Then there had been a satisfactory decrease in expenditure, showing that they were sparing no trouble to manage the hospital as economically as possible; and that was the only way they could get the public to put their trust in a hospital—to show that it was well managed. Although during the year they had treated 123 more in-patients and 1,500 more out-patients, their milk had cost £125 less, owing to new and better contracts; the cost of dressings and bandages was £42 down since they had bought larger quantities and so got better terms; their ice and mineral water account was £23 less, and that they owed to the Dock Company, through whom they now got their ice for nothing; their fuel and lighting bill was also less, as they had made a better contract for coal and coke, and electric light was also cheaper in Poplar. The cost of appeals was also down; they had advertised rather less. He thought the Committee was entitled to credit for managing the hospital with less expense, although there had been a large increase in the work done. It was extremely satisfactory that they had been able to invest £10,000, but it was an exceptional year, and they could not expect to go on at that rate. The position of the hospital financially was that they had now a certain income of about £3,000 a year from investments, the contributions of the Hospital Saturday and Sunday Funds, and the payments of patients. Their expenditure was something under £9,000, so that each year they had to raise about £6,000. They had not failed to do this for a long time, and he did not think they would so long as the public put their trust in the hospital, and that they would continue to do so long as it was well managed. The cost per bed had been reduced from £75 8s. per bed last year to £70 16s. 7d. this. In 1904 the cost per patient was £4 2s. 10d., this year it was only £3 14s. 1d.; and the cost per patient per day was down from 4s. 1¾d. to 3s. 10¾d. And, since they had plenty of working men on their Committee, it was perfectly certain that this had not been done at the cost of the comfort or well-being of the patients; they had been fed as well, nursed as well, and as well attended to. It was due to the closest scrutiny being applied to every detail. If any patient thought things were not just as they ought to be he took care to mention it to some of his friends on the Committee, and as an instance of this he might mention that at the suggestion of one of them they now had their tea in a cup and saucer, instead of a mug. (Laughter.) Their income from investments, which was now £1,700 a year, showed a striking advance since 1889, when it was only £400. (Applause.) Since those days they had rebuilt the hospital, at a cost of £70,000, they had trebled its size, and they had more than quadrupled the amount put by for a rainy day. During the past year several important changes had taken place. Their Secretary and House Governor, Colonel Feneran, who was getting old, had retired, and had been pensioned; and he had been succeeded by Mr. Rogers, who had been for four years second in command at the London Hospital. He could not close without bearing the warmest testimony to their excellent matron and nurses, and he must add a word of praise, too, for their lady dispenser, whose work had been done quite admirably.

The motion was duly seconded and adopted, the Committee was re-elected, and votes of thanks closed the proceedings.

### NATIONAL HOSPITAL FOR DISEASES OF THE HEART.

THE annual general meeting of Governors was held on March 15, when the Earl of Verulam took the chair.

The report having been read, Lord Verulam moved its adoption, and referred in feeling terms to the death of Mr. G. N. Marten, Hon. Treasurer, who had been a personal friend of his own. He was pleased to see from the report that on the whole the hospital had done well in the past year. The receipts had been £2,902 for 1905, as against £2,614 in 1904, which indicated a considerable increase, mostly due, however, to legacies, though the Hospital Sunday Fund had contributed £181 and the Hospital Saturday Fund £135. Personally, he objected very much to treating legacies as income, because they might at any time fall off; they came from capital funds and they ought to go to capital funds. They were very grateful to the kind friends who had sent gifts to the hospital and provided entertainments for the patients.

Mr. H. D. Haslewood, Hon. Treasurer, in seconding the resolution, said that the balance-sheet could not be looked upon as other than favourable. Unfortunately the time was far distant when they would be able to treat their legacies as capital. They were only able to keep things going by treating them as income. It was satisfactory to know that during the past year all the beds had been full.

Dr. C. W. Chapman, in responding to a vote of thanks to the medical staff, remarked that the report showed a great increase in the number of patients, owing a good deal to the length of time they required attention; in-patients had to become out-patients for some time. They had also chronic cases of long standing, which were only kept going by constant medical supervision, being thus enabled to go back to their families and their work and were thus kept off the rates. Many of these cases had been under medical care in other hospitals, and it was not a question of their being cured, but of enabling them to keep on at their work, sometimes for ten or fifteen years. He thought that they were inclined at that hospital to be too modest and retiring about their claims upon the public. In these days of push they were apt to be forgotten unless they put themselves more prominently forward. Their in-patients were retained a much longer time than in ordinary hospitals, otherwise their figures would make a more brilliant show. But they felt convinced they did more real good by giving more or less permanent benefit to a few than by giving temporary benefit to many. Their cases required rather expensive drug treatment, which caused the amount for drugs to be a heavy one.

The usual votes of thanks having been passed, the proceedings came to a close.

### ITALIAN HOSPITAL.

THE annual meeting of Governors was held on March 17, when Count A. de Bosdari, Councillor, Italian Embassy, presided.

The adoption of the report was moved by the Chairman, who remarked on the disappointment it had been to the late Italian Ambassador, Signor Pansa, that he had had to leave England before the meeting took place. Signor Pansa had always taken great interest in the hospital, and he felt sure that the new Ambassador, Signor Tittoni, would also show his appreciation of the work that was carried on there. For his own part it gave him great pleasure to preside on that occasion. He had known the work of the hospital for some considerable time and knew how well it was carried on.

The Very Rev. Cav. J. P. Bannin seconded the motion, and drew attention to the fact that during the past year there had been a serious diminution in annual subscriptions, owing probably to the prevalence of bad times and the recent general election. They had received £500 from King Edward's Hospital Fund for the purpose of keeping up ten extra beds, and they had also been granted £249 from the Hospital Sunday Fund, and £105 from the Hospital Saturday Fund, making a total of over £850. The sum given by the Italian Government was £100, which they could not help regarding as rather modest, in comparison with the sums received from English sources. Perhaps if this were represented in proper quarters the donation from the Italian Government might be increased.

The re-election of retiring members of the committee of management was moved by Cav. Dr. F. Naumann, who stated humorously that the House Committee had one great weakness, they suffered from attacks of "billophobia," or fear of bills, which produced great excitement at the time and was followed by severe prostration. The "rate-bacillus" was the worst feature of this disease.

A vote of thanks to H.R.H. the President of King Edward's Hospital Fund for the yearly grant of £500 was moved by Cav. Dr. M. Castañeda, the Chairman of the committee, who dwelt upon the satisfaction the visitors of the Fund had expressed with the state of the hospital on the occasion of a surprise visit they had paid. The resolution was seconded by Mr. Stuart A. Coats, and supported by the Very Rev. Cav. J. P. Bannin, who said that last year the number of patients had risen from 12,299 in 1904 to 15,575, or an increase of 3,000, without a corresponding increase in expenditure. In 1904 the expenditure had been £2,692 and in 1905 £2,715, or an increase of only £23. The average cost per bed was £38 4s. 7d., and he ventured to say that there was no hospital in London which could compare with the Italian Hospital as to the economy of its management, some averaging as high as £50, £60, or even £70 per bed. It was doubtless as a recognition of their economical administration that King Edward's Hospital Fund had given them so handsome a donation.

A vote of thanks having been passed to Count A. de Bosdari for presiding, the meeting separated.

